# m6A epigenetic signature in necrotizing enterocolitis: insights from murine to neonatal studies

**DOI:** 10.3389/fimmu.2025.1709677

**Published:** 2026-01-14

**Authors:** Yixian Chen, Qiaozhen Wei, Yuan Gan, Ba Wei, Qingmei Huang, Shuangjing Pan, Yujun Chen

**Affiliations:** 1 Department of Pediatrics, The Second Affiliated Hospital of Guangxi Medical University, Guangxi, Nanning, China; 2 Neonatology, Liuzhou Hospital of Guangzhou Women and Children’s Medical Center, Guangxi, Liuzhou, China; 3 Neonatology, Liuzhou Maternity and Child Healthcare Hospital, Guangxi, Liuzhou, China

**Keywords:** infants, m6A, MeRIP-seq, mouse, necrotizing enterocolitis

## Abstract

Necrotizing enterocolitis (NEC) is a severe intestinal infectious disease. m6A modification plays a critical role in intestinal homeostasis and infection, while its role in NEC remains unclear. This study aimed to investigate the landscape of m6A modifications in NEC, spanning from animal models to humans. This study aimed to investigate the landscape of m6A modifications in NEC, spanning from animal models to humans. In our study, m6A was widely distributed in ileal tissues, with elevated global m6A levels detected via dot blot and immunohistochemistry. qRT-PCR and western blot assays revealed increased expression of METTL3 and METTL14 in NEC mice. Subsequently, RNA-seq and MeRIP-seq were performed on ileal samples from NEC infants and controls. RNA-seq identified 2,108 differentially expressed genes (DEGs), while MeRIP-seq detected 19,242 m6A peaks in NEC and 16,318 peaks in controls, with 8,957 differential peaks. Integrative analysis of RNA-seq and MeRIP-seq datasets identified differentially m6A-modified DEGs, which were associated with leukocyte migration, adhesion, and predominantly enriched in cytokine-cytokine receptor interactions. Notably, 363 genes exhibited concurrent m6A hypermethylation and transcriptional upregulation in NEC infants, with significant enrichment in the NF-κB signaling pathway. 192 genes displayed m6A hypermethylation accompanied by transcriptional downregulation, showing significant enrichment in focal adhesion and ECM-receptor interaction pathways. This study pioneers the systematic characterization of m6A methylation patterns in NEC, offering novel insights into m6A-mediated molecular mechanisms driving NEC pathogenesis.

## 1 Introduction

Necrotizing Enterocolitis (NEC) is a severely life-threatening disease for premature infants. The incidence rate of NEC in extremely low birth weight premature infants is approximately 7% (1). Approximately one-tenth of neonatal deaths can be attributed to NEC. Among the surviving children, 25%-61% and 15%-35% of the children have residual neurodevelopmental disorders and intestinal dysfunction respectively (2). Despite the continuous increase and in-depth research on NEC, the pathogenesis of NEC remains incompletely understood.

Epigenetics bridges environmental factors with heritable gene expression alterations through reversible modifications of nucleotides or associated proteins, without altering DNA sequences. External factors including nutrition, infection, medication, and physiological stress regulate these processes (3, 4). N6-methyladenosine (m6A), the most prevalent RNA modification in mammals, is characterized by the methylation at the sixth nitrogen atom of adenosine. This modification is under dynamic regulation by “writers” (including METTL3, METTL14, WTAP, and METTL16) and “erasers” (such as FTO, ALKBH5). The m6A-modified RNA bases are recognized by “readers” (e.g., YTHDF1-2, YTHDC1-2, IGF2BP1-3, HuR, EIF3), which regulate mRNA translation, degradation, and stability. m6A has an impact on cellular growth, immunity, tissue repair, and disease processes including cancer, infections, immune disorders, and cardiovascular and neurological diseases (5).

Dysregulation of m6A regulators (writers, erasers, readers) impacts intestinal barrier function, microbiota, and immune homeostasis (6). In inflammatory bowel disease (IBD), elevated global m6A levels are observed in ulcerative colitis and Crohn’s disease tissues compared to controls, with altered expression of METTL3, FTO, METTL14, and HuR (7). Notably, METTL3 and FTO are upregulated during active IBD but downregulated in remission (8). Mechanistically, METTL3 promotes toll-like receptor 4 (TLR4) mRNA translation and stability via m6A modification, enhancing TLR4 protein expression and neutrophil activation through CXCR2-mediated bone marrow release in lipopolysaccharide (LPS)-induced endotoxemia (9). METTL14 deficiency induces apoptosis in colonic stem cells, disrupts mucosal barrier integrity, and exacerbates colitis by destabilizing nuclear factor kappa B inhibitory protein (NFKBIA) mRNA and dysregulating NF-κB pathways (10). METTL3 knockdown reduces expression of tight junction proteins (ZO-1, Occludin, Claudin-3), impairing intestinal barrier function (11). Conversely, IGF2BP1 and HuR stabilize mRNAs encoding Occludin, JAM-1, Claudin-1, Claudin-3, and E-cadherin, thereby enhancing translation and maintaining mechanical barrier integrity (12). Collectively, these findings demonstrate that m6A modification plays critical regulatory roles in intestinal inflammation, barrier integrity maintenance, and cell death. As a multifactorial infectious intestinal disorder (13), the precise role of m6A in NEC pathogenesis remains to be fully elucidated.

Advances in high-throughput sequencing and bioinformatics have enabled genome-wide mapping of m6A modifications across species (14), diseases (15), and tissues via methylated RNA immunoprecipitation sequencing (MeRIP-seq). In this study, we first investigated RNA m6A modifications in a neonatal mouse NEC model, revealing elevated global m6A level and imbalanced expression of m6A regulators. Subsequently, ileal tissues from NEC patients underwent transcriptome sequencing (RNA-seq) and MeRIP-seq to delineate transcriptomic signatures and m6A modification landscapes. Integrated analysis identified differentially m6A-modified DEGs and related pathways. This work establishes a foundation for unraveling m6A role in NEC and identifying novel therapeutic targets.

## 2 Materials and methods

### 2.1 Animals

Four pregnant C57BL/6J mice at 18-19-day gestation were obtained from the Experimental Animal Center of Guangxi Medical University. Animals were maintained under specific pathogen-free (SPF) conditions with a 12-h light/dark cycle and ad libitum access to food/water at the Experimental Animal Center of Guangxi Medical University. Following natural delivery, newborn pups derived from mixed-sex litters were maternally suckled for 72 hours and then randomly allocated to either a control group (n=6) or an NEC model group (n=19). All procedures were performed in compliance with protocols approved by the Institutional Animal Care and Use Committee (IACUC) of Guangxi Medical University [2023-KY (0801)].

### 2.2 Patients

Ileal tissues were collected from infants undergoing surgery at Liuzhou Hospital of Guangzhou Women and Children’s Medical Center and the Second Affiliated Hospital of Guangxi Medical University. NEC was confirmed by pathological diagnosis, with necrotic ileal tissues resected from NEC infants. Control tissues were non-necrotic ileal samples from infants with congenital intestinal stenosis, atresia, or ileostomy. Exclusion criteria included spontaneous intestinal perforation, chromosomal abnormalities, major malformations, and genetic metabolic disorders. Specimens were cut into ≤0.5 cm segments, immediately submerged in 5× volume of RNA solid™ tissue stabilization solution (G3019, Servicebio, China), stored at 4°C overnight, and transferred to −80°C for long-term storage. All participants provided written informed consent in accordance with the Declaration of Helsinki. Ethical approval was obtained from the respective institutional review boards [approval numbers: 2022–069 and 2023-KY(0801)].

### 2.3 Establishment of animal model

The NEC murine model was established in 4-days-old mice using “formula + hypoxia + hypothermia + lipopolysaccharide (LPS)” (16). Briefly, mice were fed with high-osmolarity formula milk (Nestle Infant Formula, 750 Osm/L), 5 times a day (at 8:00, 12:00, 16:00, 20:00, and 24:00). Each feeding session involved a volume of 50 μl/g, and the gavage process lasted for 1–2 minutes. Mice received hypoxia stress (breathing 5% oxygen + 95% nitrogen for 10 min), followed by cold stress (4°C for 15 min) for 3 times a day (at 10:00, 18:00, and 22:00). Moreover, the mice were administered LPS (L2880, Sigma, America) via an intragastric tube. The dosage was 10 mg/kg, with a concentration of 1 μg/μl, and this was done once daily for 4 consecutive days. The neonatal mice in the control group were housed with their mothers and were breastfed. The only regular procedure performed on them was daily weighing, without any other interventions.

### 2.4 Tissue collection and NEC injury assessment

Following model establishment, the pups were euthanized by decapitation, ileal tissues were harvested. A 1-cm segment of terminal ileum proximal to the cecum was excised for histological evaluation. Remaining ileal samples were immediately snap-frozen in liquid nitrogen and stored at −80°C for downstream analyses. Excised tissues for histology were fixed in 4% paraformaldehyde (PFA) for 72 hours, dehydrated through a graded ethanol series, embedded in paraffin, and sectioned into 4-μm-thick slices. Then the sections were stained with Hematoxylin and Eosin (HE) staining according to the manufacturer’s instructions. An established scoring criterion was utilized to conduct the pathological injury assessment (17), with two independent pathologists blinded to the study. Mice with a pathological injury score >2 were classified as having NEC.

### 2.5 Immunohistochemical staining

Paraffin-embedded sections with a thickness of 4 µm were deparaffinized, and antigen retrieval was performed. Subsequently, the sections were blocked with a solution containing 1% normal goat serum and H_2_O_2_ for 20 minutes. After blocking, the sections were incubated overnight at 4°C with a rabbit anti-FXR polyclonal antibody. Following the primary antibody incubation, the sections were incubated with a horse - radish peroxidase (HRP) - coupled secondary antibody at room temperature for 20 minutes. For each tissue sample, three random fields of view were selected and imaged under a microscope at a magnification of 400×. The captured images were then analyzed using ImageJ software. The integrated optical density (IOD) of each field of view was calculated for subsequent statistical analysis.

### 2.6 RNA extraction

Total RNA was extracted from the intestine of mice using the total RNA extraction kit (RC112-01, Vazyme, China). Total RNA was extracted from the intestine of human using Trizol reagent according to the manufacturer’s instructions. The integrity and concentration of extracted RNAs were detected using an Agilent 2100 bioanalyzer (Agilent) and simpliNano spectrophotometer (GE Healthcare), respectively.

### 2.7 Quantitative real-time polymerase chain reaction analysis

Total RNA was reverse-transcribed into cDNA using a reverse-transcription kit (R323-01, Vazyme, China). The products were directly used for qRT-PCR with specific primers (**Table 1**) synthesized by Shanghai Sangon Biotech Co. The reaction mix was: 2×RealStar Fast SYBR qPCR Mix (Low ROX) 10 µL, each primer 0.5 µL, cDNA 1 µL after 1:10 dilution, and the final volume was 20 µL. The conditions of PCR were 2 min at 95 °C, followed by 40 amplification cycles of 10 s at 95 °C and 30 s at 60°C. Shrimp β-actin was used as an internal reference. 2^−(△△Ct)^ method determined the gene expression levels.

**Table 1:** Sequence of the primers.

Gene	Primer type	Sequence
Mettl3	Forward	TCCATCCGTCTTGCCATCTCTAC
	Reverse	CCTCGCTTTACCTCAATCAACTCC
Mettl14	Forward	AGCAGACATAGAAGCCTTTGACATC
	Reverse	AATATCATCCCAGGTCCAGCATTTC
WTAP	Forward	AGAACATTCTTGTCATGCGGCTAG
	Reverse	CACACTCGGCTGCTGAACTTG
FTO	Forward	GGAGGAACGAGAGCGGGAAG
	Reverse	GCTGCCACTGCTGATAGAACTC
β-actin	Forward	ACTGCCGCATCCTCTTCCTC
	Reverse	AACCGCTCGTTGCCAATAGTG

### 2.8 Dot blot analysis for mouse ileal m6A level

Mouse intestinal RNA samples were adjusted to concentrations of 100 ng/μl and 200 ng/μl. The RNA was denatured by heating at 95°C for 3 minutes. A total of 1.5 μl of each diluted RNA sample was spotted onto a nylon membrane. Immediately after spotting, UV cross-linking (254 nm, 120 mJ/cm²) was performed for 1 hour. The membrane was then washed with TBST for 5 minutes. Next, it was stained with 0.02% methylene blue in water at room temperature for 5 minutes. After staining, the membrane was rinsed with TBST until the RNA spots became clearly visible, and then photographed for documentation.

For immunodetection, the membrane was blocked with 5% skimmed milk in TBST for 2 hours at room temperature. It was then incubated overnight at 4°C with a rabbit anti-m6A primary antibody (Proteintech, 1:5000). After three washes with TBST (10 minutes each), the membrane was incubated with a horseradish peroxidase (HRP) - conjugated goat anti-rabbit secondary antibody (Proteintech, 1:15000) for 1 hour at room temperature. Finally, the immunoreactive signals were visualized using an enhanced chemiluminescence (ECL) kit (BL520A, Biosharp, China) and detected with a chemiluminescent imaging system.

### 2.9 Western blot

Total protein was extracted using RIPA lysis buffer supplemented with protease inhibitors. Protein concentrations were quantified using a BCA assay kit (P0012S, Beyotime, China). 50 μg protein were resolved by 10% SDS-PAGE and subsequently transferred onto 0.22 μm PVDF membranes. Membranes were blocked with 5% skimmed milk for 2 h at room temperature, then incubated overnight at 4°C with the following primary antibodies: anti-METTL3 (Abcam, 1:1000), anti-METTL14 (Proteintech, 1:5000), anti-WTAP (Proteintech, 1:5000), anti-FTO (Proteintech, 1:5000), and anti-β-actin (Proteintech, 1:2000) as loading control. After TBST washes (3×10 min), membranes were incubated with species-matched secondary antibodies (Invitrogen, 1:30,000) for 1 h at room temperature. Following additional TBST washes (3×10 min), protein signals were detected using an Odyssey^®^ CLx Imaging System (LI-COR) with dual-color infrared scanning.

### 2.10 Transcriptome sequencing (RNA-seq)

Messenger RNA was purified from total RNA using poly-T oligo-attached magnetic beads. After fragmentation, the first strand cDNA was synthesized using random hexamer primers followed by the second strand cDNA synthesis. The library was ready after end repair, A-tailing, adapter ligation, size selection, amplification, and purification. The library was checked with Qubit and real-time PCR for quantification and bioanalyzer for size distribution detection.

After library quality control, different libraries were pooled based on the effective concentration and targeted data amount, then subjected to Illumina sequencing. The basic principle of sequencing is “Sequencing by Synthesis”, where fluorescently labeled dNTPs, DNA polymerase, and adapter primers are added to the sequencing flow cell for amplification. As each sequencing cluster extends its complementary strand, the addition of each fluorescently labeled dNTP releases a corresponding fluorescence signal. The sequencer captures these fluorescence signals and converts them into sequencing peaks through computer software, thereby obtaining the sequence information of the target fragment.

### 2.11 Methylated RNA immunoprecipitation sequencing

The RNA m6A was sequenced by MeRIP-seq at Novogene (Beijing, China). Briefly, a total of 2 µg RNAs were extracted from tissues. Fragmented RNA (~100 nt) was incubated for 2 hr at 4°C with anti-m6A polyclonal antibody (Synaptic Systems) in the immunoprecipitation experiment. Then, immunoprecipitated RNAs or Input was used for library construction with Ovation SoLo RNA-Seq System Kit (NuGEN). The library preparations were sequenced on an Illumina platform with a paired-end read length of 150 bp according to the standard protocols.

### 2.12 RNA-seq and MeRIP‐seq data analysis

Raw data (raw reads) of fastq format were firstly processed through fastp software. In this step, clean data (clean reads) were obtained by removing reads containing adapter, reads containing ploy-N and low quality reads from raw data. FeatureCounts v1.5.0-p3 was used to count the reads numbers mapped to each gene. Index of the reference genome was built using Hisat2 (v2.0.5) and paired-end clean reads were aligned to the reference genome using Hisat2 (v2.0.5). In RNA-seq, FPKM of each gene was calculated based on the length of the gene and reads count mapped to this gene. Differential expression analysis for two groups was performed using the DESeq2 R package (1.20.0). DESeq2 provides statistical programs for determining differential expression in digital gene expression data using models based on negative binomial distribution. For MeRIP‐seq data, after mapping reads to the reference genome, exomePeak R package (version 2.16.0) was used for the m6A peak identification in each anti-m6A immunoprecipitation group with the corresponding input samples serving as a control, and q-value threshold of enrichment of 0.05 was used for all data sets. The m6A-enriched motifs of each group were identified by HOMER (version 4.9.1). In the peak calling result, each peak was corresponding on gene, in which the peak was located in its exon. These genes were supposed as peak related genes. Gene Ontology (GO) enrichment analysis of differentially expressed genes was implemented by the clusterProfiler R package, in which gene length bias was corrected. GO terms with corrected P-value less than 0.05 were considered significantly enriched by differential expressed genes or differential peak related genes. We used clusterProfiler R package to test the statistical enrichment of differential expression genes or differential peak related genes in Kyoto Encyclopedia of Genes and Genomes (KEGG) pathways.

### 2.13 Statistical analysis

All statistical analyses were performed using SPSS 26.0. Data were expressed as mean ± standard deviation ( $\bar{x}$ ± s) and differences were assessed by a two‐tailed Student’s t test. And all bioinformatics analyses were performed using Fisher’s exact test. GraphPad Prism 10.0 was used to create the graphs, and p values of 0.05 were considered statistically significant.

## 3 Results

### 3.1 Evaluation of the murine NEC model

**Figure 1:**
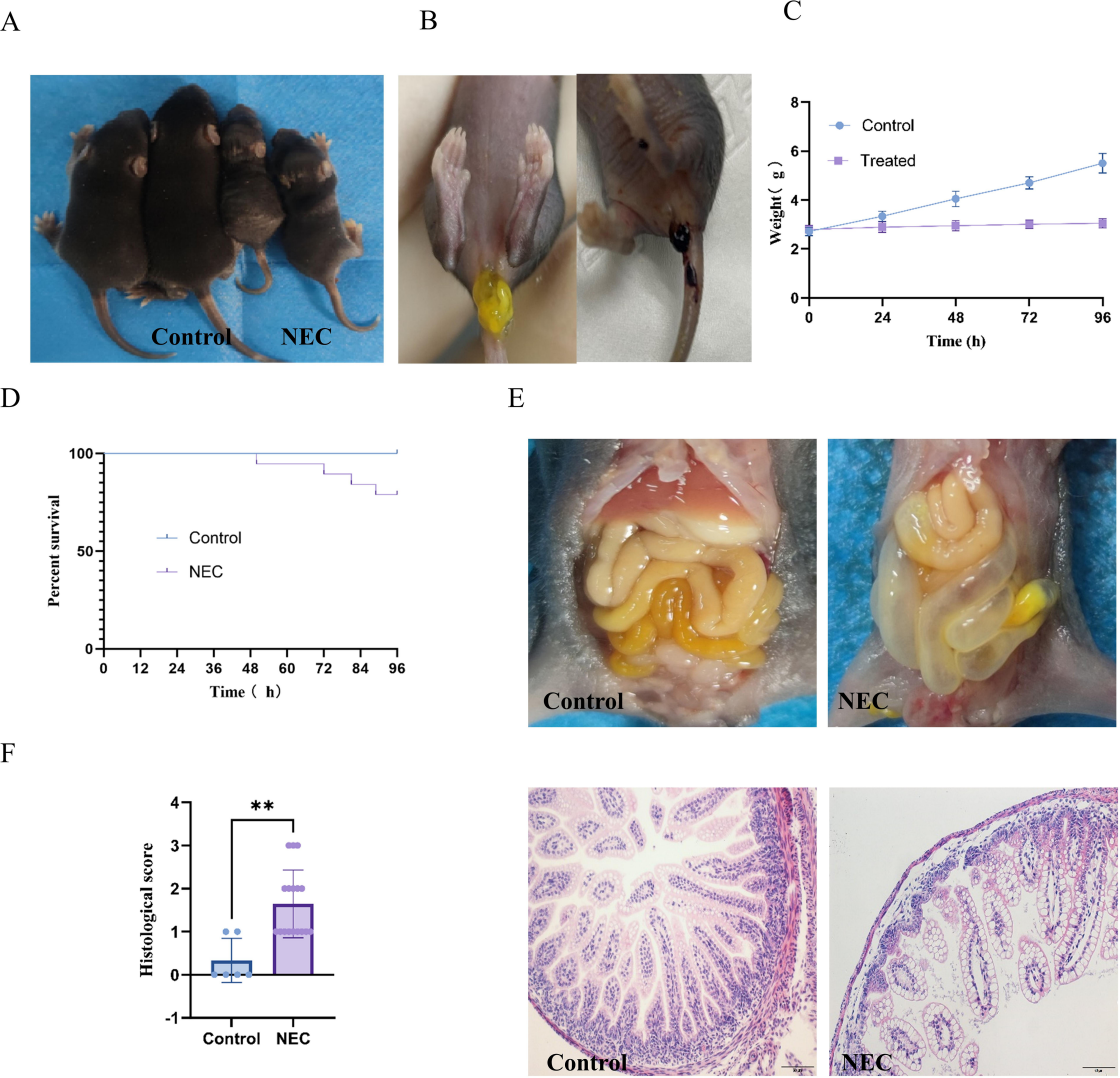
Phenotypic and histological characterization of the murine NEC model. **(A)** Gross appearance of control and NEC mice. **(B)** NEC-specific stool abnormalities: foam stool (left) and hemorrhagic stool (right). **(C)** Temporal changes in body weight during intervention. **(D)** Kaplan-Meier survival curves of NEC and control mice. **(E)** Abdominal distension in NEC mice compared to controls. **(F)** HE-stained ileal sections and histological scoring of intestinal injury. ** indicates p< 0.01.

Mice in the NEC group exhibited poor general status, including reduced spontaneous activity, diminished response to stimuli, exhibited coarse and dull fur, along with dry, rough skin showing reduced elasticity (**Figure 1A**), coarse and dull fur, loose/foamy stools, occasional melena (**Figure 1B**), and impaired weight gain (**Figure 1C**). The mortality rate in the model group was 21.05% (4/19), while no deaths occurred in the control group (**Figure 1D**). The intestinal tissue of control mice appeared pale yellow and elastic, whereas the gastrointestinal tract of model mice was rigid, inelastic, with marked intestinal distension, intestinal wall thinning and fragility (**Figure 1E**).

HE staining revealed intact ileal architecture in controls. In contrast, model mice displayed thinning of the pink striated border, villous shortening, disorganized epithelial cell arrangement, edema, detachment of epithelial cells, villous edema, separation of submucosal and lamina propria layers, partial villous necrosis, inflammatory cell infiltration, and vascular congestion. Histological scores of ileal tissues were 0–1 in controls, while model group scores varied significantly (**Figure 1F**). Mice with scores ≥2 were included in subsequent analyses.

### 3.2 The m6A modification in NEC mice

**Figure 2:**
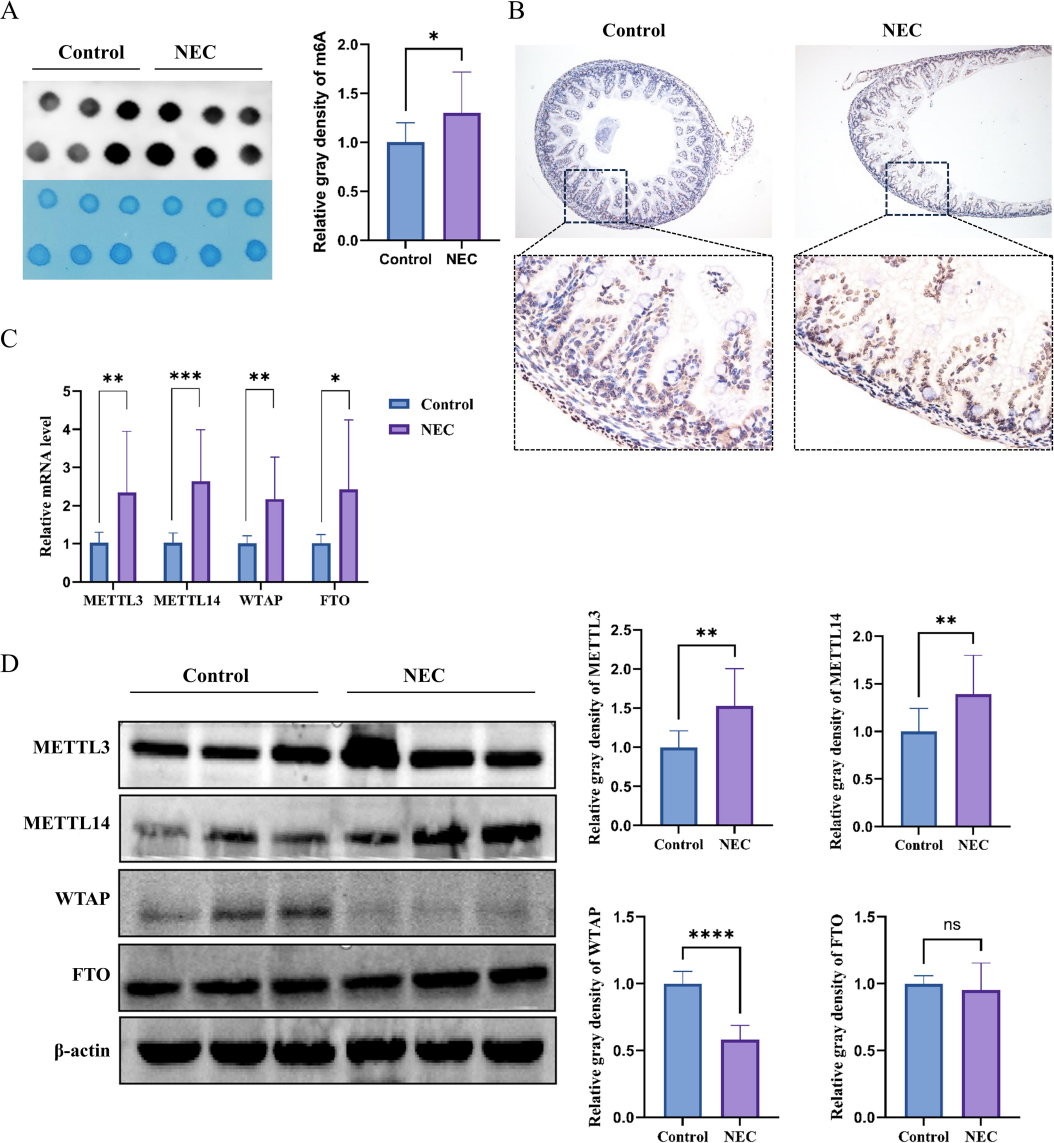
m6A modification in intestinal tissue from mouse. **(A)** Dot blot showed the overall level of m6A in two groups (top) and methylene blue staining as a control (bottom) (n=6). **(B)** IHC shows the localization and expression of m6A modification in intestine. Brown staining indicates the presence of m6A modification. **(C)** The mRNA expression levels of METTL3, METTL14, WTAP and FTO (n=5). **(D)** The protein expression levels of METTL3, METTL14, WTAP and FTO (n=6). * indicates p< 0.05, ** indicates p< 0.01, ***indicates p< 0.001, and **** indicates p< 0.0001.

Dot blot showed that the overall level of RNA m6A in the ileal tissues of NEC mice was significantly higher than that in the control group (**Figure 2A**). The IHC results demonstrated that m6A modification, mainly located in the cell nucleus, was widely present in multiple cell types of the mouse ileal tissues (**Figure 2B**).

The mRNA levels of METTL3, METTL14, WTAP, and FTO in the intestinal tissues of NEC mice increased significantly (**Figure 2C**). The protein levels of METTL3 and METTL14 increased significantly, while the protein level of WTAP decreased, and the protein level of FTO showed no significant difference (**Figure 2D**). Based on the above research results, it is suggested that there is an imbalance in the expression of regulatory factors for m6A modification in NEC, and cause increase in the overall m6A level in the ileal tissues of NEC.

### 3.3 Distribution characteristics of m6a peaks in nec infants

**Figure 3:**
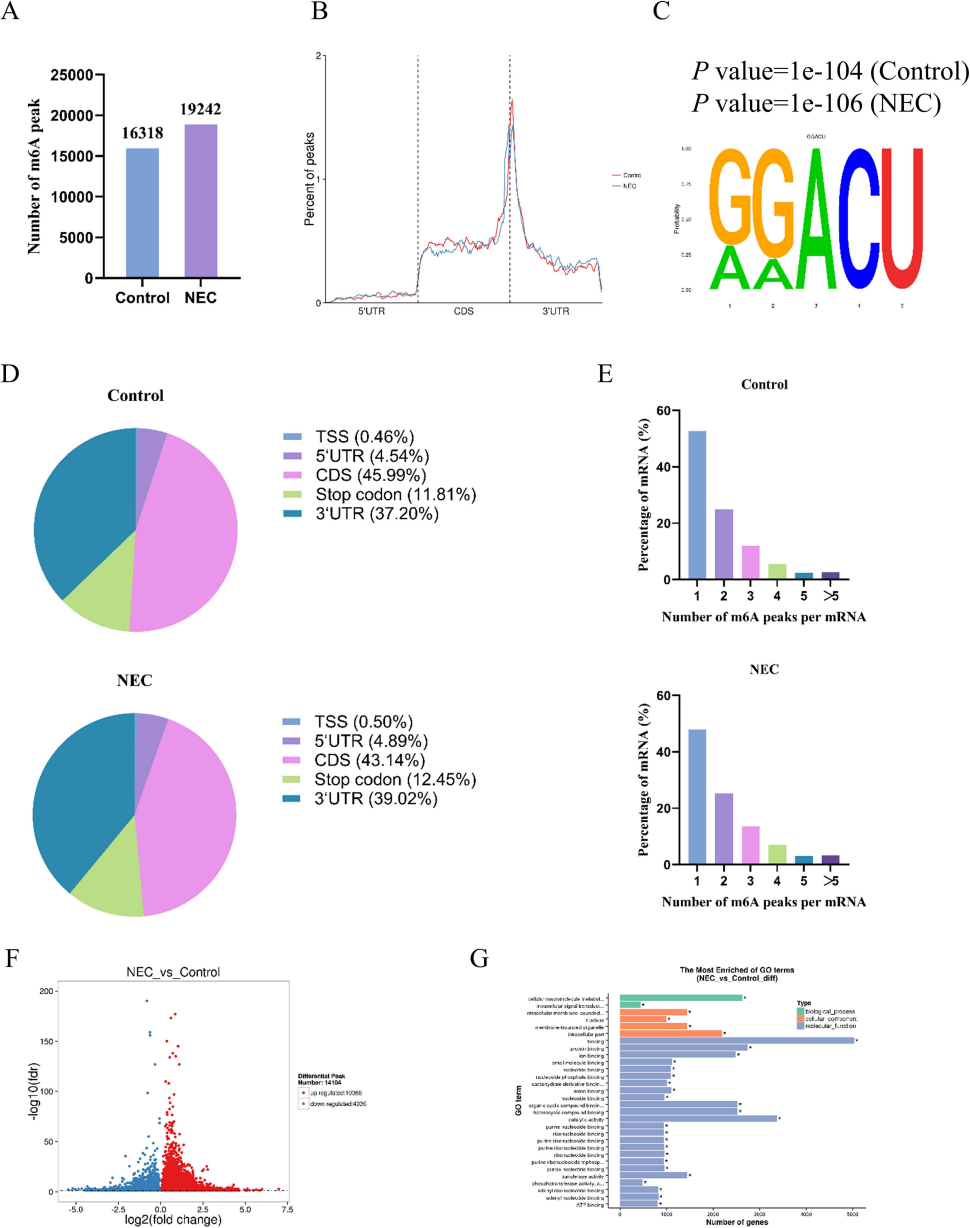
m6A modification in intestinal tissue from infants. **(A)** Number of peaks in two groups. **(B)** The distribution of peak in different regions of mRNA (5’UTR, CDS, 3’UTR). **(C)** The most confidential sequence with m6A in both groups. **(D)** The distribution map of peaks in five transcriptional functional regions. **(E)** Number of peaks on mRNA. **(F)** Volcano map of differential peaks, upregulated genes are represented by red dots, downregulated genes are represented by blue dots. **(G)** GO enrichment analysis of genes with differential peaks, * indicates Padj value<0.05.

There were 8 samples from NEC (NEC group)and non-NEC infants (control group) (n=4, respectively) were used for RNA-seq and MeRIP-seq. The clinical features of the participants are shown in **Table 2**. MeRIP-Seq identified 19,242 m6A peaks representing 9,315 genes of in the NEC group. In the control group, 16,318 m6A peaks were identified and represented 8,520 genes, suggesting that the overall m6A modification level in the ileal tissues of the NEC group was higher than that in the ileal tissues of the control group (**Figure 3A**).

**Table 2:** Basic information of infants included in the study.

Sample	GW (weeks)	BW (g)	Gender	Diagnosis	Age	Sample type
NEC1	27^+4^	950	male	NEC	76 days	ileum
NEC2	27^+6^	1290	male	NEC	23 days	ileum
NEC3	31^+5^	1150	male	NEC	37 days	ileum
NEC4	27	920	male	NEC	5 days	ileum
Control1	28^+1^	1700	male	ileostomy	3 months	ileum
Control2	33	1700	male	ileostomy	3 months	ileum
Control3	30^+1^	1300	male	congenital intestinal atresia	3 days	ileum
Control4	35	2100	male	congenital intestinal stenosis	4 days	ileum

Age: Age at specimen collection.

m6A modifications are not randomly enriched on mRNAs. We divided transcript into three segments, the 5’ untranslated region (5’ UTR), the coding sequence (CDS), and the 3’ untranslated region (3’ UTR), and then counted the distribution of peaks in these segments. The vast majority of peaks were enriched in CDS region near the 3’ UTR region (**Figure 3B**). Motif analysis showed that when the recognition length was 5 bases, the most confidential sequence in both groups was GGA(m^6^A)CU (**Figure 3C**). **Figure 3E** shows the number distribution of m6A peaks on mRNAs in both groups. Of these, 52.72% of genes in the control group harbored a single peak on their mRNAs, and 47.84% of genes in the NEC group contained a single peak. To further evaluate the distribution of peaks on mRNA transcript, we divided mRNA transcript into CDS, 5’ UTR, 3’ UTR, transcription start sites (TSS) region, and stop codon region according to functional domains. The result shown that the proportion of peaks in the CDS region was the highest in both the control group and the NEC group, which were 45.99% and 43.14% respectively. Followed by the 3’ UTR and stop codon regions, which were 37.2% and 11.81% in the control group, and 39.02% and 12.45% in the NEC group respectively (**Figure 3D**).

### 3.4 Function analysis of genes associated with differential m6A peaks

14,104 differential peaks between the NEC group and the control group, corresponding to 8,957 genes. Compared with the control group, 10,068 peaks were upregulated in the NEC group, corresponding to 6,869 genes, and 4,036 peaks were downregulated, corresponding to 3,357 genes. Among them, 1,269 genes had both upregulated and downregulated peaks (**Figure 3F**).

To understand the functions of these genes, we conducted enrichment analysis on the genes of differential peaks. Through GO function enrichment analysis (**Figure 3G**), we found that the genes of differential peaks were mostly enriched in cellular macromolecule metabolic processes, intracellular signal transduction, small GTPase - mediated signal transduction, and cellular metabolic processes. They were also related to cellular components such as intracellular membrane-bounded organelle, nucleus, membrane-bounded organelle, intracellular parts, and intracellular organelles; and to molecular functions such as binding, protein binding, ion binding, small molecule binding, and nucleotide binding.

### 3.5 Differentially expressed genes

**Figure 4:**
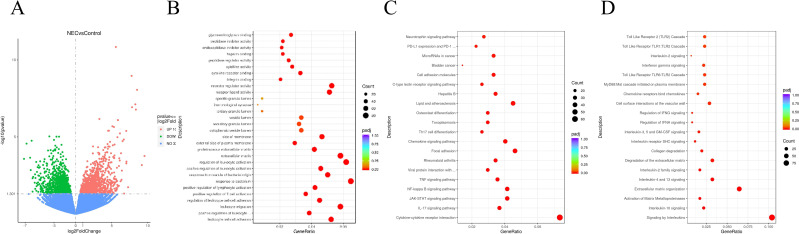
DEGs from RNA-seq. **(A)** The volcano plot distribution of DEGs. Upregulated genes are represented by red dots, downregulated genes are represented by green dots, and blue dots represent non DEGs. **(B)** GO function, **(C)** KEGG and **(D)** Reactome enrichment analysis of DEGs.

Based on gene expression levels in RNA-seq, we identified differentially expressed genes (DEGs) between NEC and control groups. A total of 2,108 DEGs were detected, including 1,070 upregulated and 1,038 downregulated genes. Statistical results of DEGs are shown in **Figure 4A**, with volcano plots visualizing DEG distribution. GO function enrichment analysis of DEGs (**Figure 4B**) highlighted significant enrichment in biological processes, including leukocyte cell-cell adhesion, positive regulation of leukocyte adhesion, leukocyte migration, and leukocyte adhesion regulation. Cellular components were primarily associated with extracellular matrix, proteinaceous extracellular matrix, extracellular plasma membrane regions, and cytoplasmic membrane compartments. Molecular functions were significantly linked to receptor ligand activity, receptor regulatory activity, integrin binding, and cytokine receptor binding.

To further characterize DEGs functions, pathway enrichment analysis was performed using KEGG and Reactome databases. KEGG enrichment analysis showed DEGs significantly enriched included cytokine-cytokine receptor interaction, interleukin-17 (IL-17) signaling pathway, JAK-STAT signaling pathway, and NF-κB signaling pathway (**Figure 4C**). Reactome enrichment analysis showed enrichment in signaling by interleukins, IL-10 signaling, activation of matrix metalloproteinases, and extracellular matrix organization (**Figure 4D**).

### 3.6 Integrated analysis of MeRIP-seq and RNA-seq data

**Figure 5:**
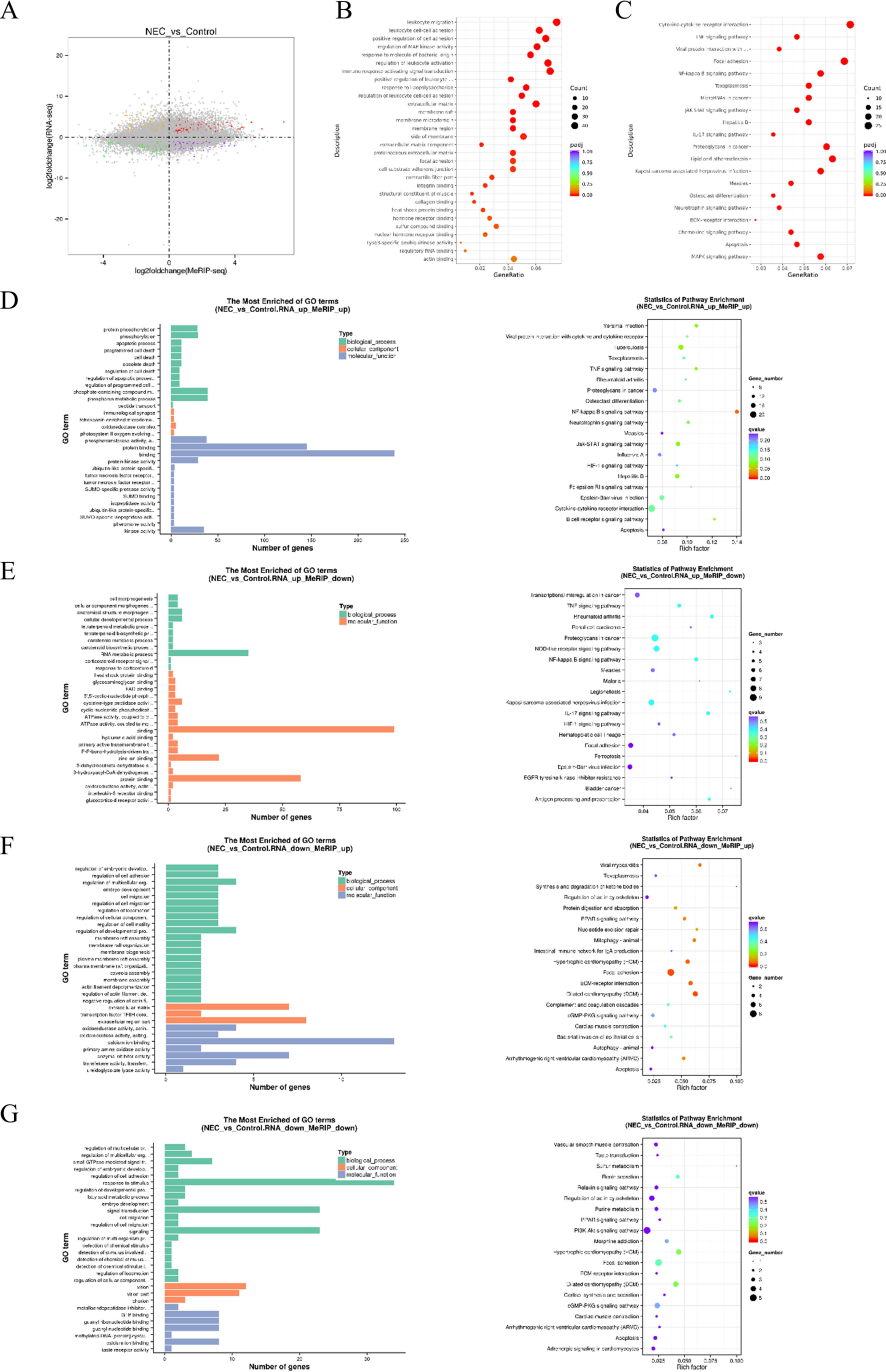
Integrated analysis of DEGs and differential m6A peaks related genes. **(A)** Four quadrant diagram, with colored dots represent the intersection genes of differential m6A peak related genes and DEGs. **(B)** GO function and **(C)** KEGG enrichment analysis of differentially m6A-modified DEGs. **(D–G)** GO function and KEGG enrichment analysis of distinct categories of differentially m6A-modified DEGs.

Single MeRIP-seq lacks information on the expression levels of the regulated mRNAs, while RNA-seq cannot reveal the effect of m6A on gene expression. To further clarify which genes have undergone changes in both m6A methylation and transcription levels, and thereby explain the regulatory role of m6A in gene expression, this study conducted a combined analysis of the RNA-seq and m6A-seq results. The four-quadrant plots showed the relationship between m6A methylation and mRNA expression (**Figure 5A**). The results showed that 363 genes were m6A hypermethylation and transcriptional upregulation (RNA_up_MeRIP_up), 192 genes were m6A hypermethylation and transcriptional downregulation (RNA_down_MeRIP_up), 150 genes were m6A hypermethylation and transcriptional upregulation (RNA_up_MeRIP_down), and 136 genes were m6A hypomethylation and transcriptional downregulation (RNA_down_MeRIP_down). These genes are referred to as differentially m6A-modified DEGs.

GO and KEGG pathway enrichment analysis were performed on differentially m6A-modified DEGs. The results demonstrated that these genes were associated with biological processes such as leukocyte migration, leukocyte cell-cell adhesion, and positive regulation of cell adhesion (**Figure 5B**), and were significantly enriched in pathways including cytokine-cytokine receptor interaction, TNF signaling pathway, and viral protein interaction with cytokines and cytokine receptor (**Figure 5C**). To further investigate the potential pathways involved in distinct categories of differentially m6A-modified DEGs (RNA_up_MeRIP_up, RNA_down_MeRIP_up, RNA_up_MeRIP_down, RNA_down_MeRIP_down), GO and KEGG (**Figures 5D–G**) pathway enrichment analysis were conducted separately for each group. The results revealed that RNA_up_MeRIP_up genes were potentially linked to the NF-κB signaling pathway, while RNA_down_MeRIP_up genes were enriched in pathways such as dilated cardiomyopathy (DCM), focal adhesion, and ECM-receptor interaction.

### 3.7 PPI network construction and identification of hub genes

**Figure 6:**
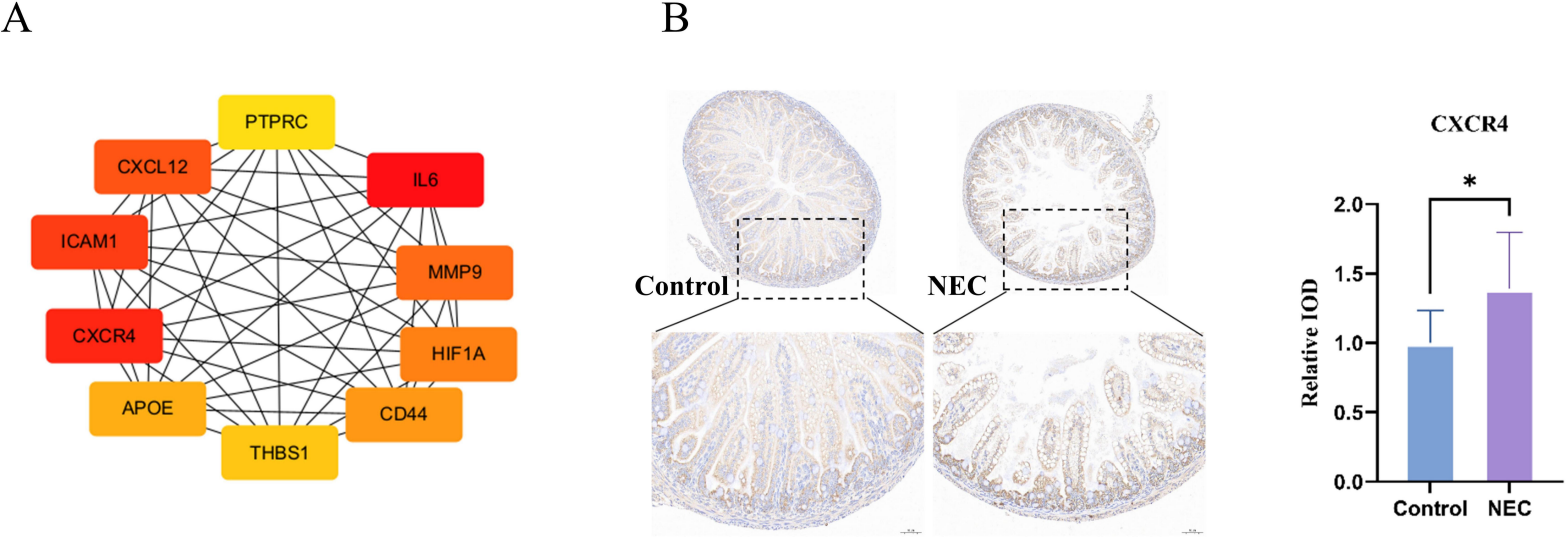
Hub genes of DEGs with differential m6A modifications. **(A)** List of top 10 hub genes. The color gradient reflects the gene ranking. Red represents the most core genes, and the color gradually fades to yellow. **(B)** IHC staining of CXCR4 in mice ileum (n=4). Scale bar, 50um. IOD: integral optical density. * indicates p< 0.05.

Using the STRING database, we constructed a protein-protein interaction (PPI) network for differentially m6A-modified DEGs. Employing the Maximal Clique Centrality (MCC) algorithm in the cytoHubba plugin, we identified the top 10 hub genes (**Figure 6A**), including IL6 (RNA_up_MeRIP_down), CXCR4 (RNA_up_MeRIP_up), ICAM1 (RNA_up_MeRIP_down), CXCL12 (RNA_down_MeRIP_up), MMP9 (RNA_up_MeRIP_up), HIF1A (RNA_up_MeRIP_down), CD44 (RNA_up_MeRIP_up/down), APOE (RNA_down_MeRIP_up), THBS1 (RNA_up_MeRIP_up/down), and PTPRC (RNA_up_MeRIP_up). These findings highlight the critical role of these genes in m6A-mediated NEC pathogenesis.

### 3.8 CXCR4 expression in NEC

Given the elevated global m6A levels in NEC intestinal tissues and the fact that CXCR4 is a differentially expressed gene with increased m6A modification and is closely associated with cytokines, cytokine receptors, and inflammatory pathways, we hypothesized that CXCR4 may play a crucial role in the m6A-mediated pathogenesis of NEC. Immunohistochemistry (IHC) staining revealed CXCR4 localized primarily on cell membranes (**Figure 6B**), particularly in crypt cells, with significantly higher expression in NEC tissues. Collectively, these results indicate that m6A modification may activate inflammatory responses in NEC by regulating the CXCR4 signaling pathway.

## 4 Discussion

We adopted a sequential strategy to explore m6A’s role in NEC. Given the hypothesis’s exploratory nature and the scarcity of clinical samples, we first employed a mouse model to robustly establish global m6A dysregulation. This provided the rationale for targeted m6A sequencing on limited human NEC specimens, which pinpointed CXCR4 as a key candidate. Finally, we validated this target in the mouse model, confirming its congruent upregulation. This step verified the model’s clinical relevance, establishing it as an essential platform for future mechanistic studies of the “m6A-CXCR4 axis”, investigations that would not be feasible in patients.

As the most abundant modification on mRNA, m6A modification is primarily regulated by methyltransferases and demethylases. Methyltransferases form the methyltransferase complex (MTC) to catalyze m6A formation. METTL3, an S-adenosylmethionine (SAM)-binding protein, transfers methyl groups from SAM, while METTL14 stabilizes MTC structure through 1:1 complex formation with METTL3. WTAP recruits METTL3-METTL14 to nuclear regions, enabling MTC to methylate adenine residues on RNA. Demethylases remove m6A modifications, and modified RNA sites are recognized by specific reader proteins to regulate mRNA splicing, nuclear export, translation efficiency, and stability (6, 18). Studies have shown that METTL3/WTAP expression positively correlates with human m6A levels, while METTL3/METTL14 correlate with murine m6A levels. ALKBH5 expression negatively correlates with m6A signals in both species, but no association exists with FTO (5). Our study confirmed widespread m6A modification in murine ileal tissues across multiple cell types, with significantly increased m6A levels and METTL3/METTL14 protein expressions in NEC mice tissues. Unchanged FTO expression suggests that m6A regulation in NEC mice ileum may primarily depend on METTL3/METTL14. Previous research indicated LPS promotes METTL3/METTL14 expression in intestinal epithelial cells, with METTL3 exacerbating LPS-induced inflammation, apoptosis, and oxidative stress while inhibiting antioxidative activity (19). (20) linked increased m6A methylation to colitis pathogenesis, showing melatonin reduces colonic methylation via MTNR1B-mediated METTL3 inhibition. However, conflicting findings highlight the complexity of m6A-mediated inflammation regulation. For example, LPS-stimulated macrophages exhibit decreased global m6A and METTL3 expression, with METTL3 knockdown upregulating pro-inflammatory cytokines (TNF-α, IL-6, NO) through NOD1/RIPK2 mRNA stabilization (21), indicating cell-specific m6A functions. In this study, we observed elevated WTAP mRNA levels alongside decreased protein levels in NEC samples. This discrepancy may be attributed to post-transcriptional regulation. TLR4, a key mediator in NEC pathogenesis, can be activated by pathogens to induce IFN-I expression. Notably, Ge et al. (22) demonstrated that IFN-I signaling activation triggers WTAP degradation via the K48-linked ubiquitin-proteasome pathway. This mechanism provides a plausible explanation for the observed uncoupling between WTAP transcription and protein expression.

In recent years, more and more studies have used MeRIP seq technology to analyze the m6A modification characteristics of the whole gene transcriptome, and analyzed the m6A modification of adult tissue transcriptome. It was found that m6A modification has conservation and organ tissue specificity, and can regulate gene expression. Among them, widely expressed genes are more likely to be regulated by m6A, and m6A modification has a certain distribution pattern, mostly enriched in the 3’UTR and near the stop codon regions, with conserved sequence RRACH motifs (R=G or A); H=A, C or U) (23). In this study, we found that the total m6A modification level in human NEC ileal tissue increased, consistent with the results of mouse NEC. The m6A modification in human ileal tissue had the highest proportion in the CDS region, mainly enriched near the 3’UTR and stop codon regions, which is consistent with the previous studies (23). (24) performed whole transcriptome m6A modification detection on the brain and gut, identifying 10151 conserved peaks in 6248 genes of brain tissue and 7074 m6A conserved peaks in 4797 genes of gut tissue. Further comparison revealed that only 13 genes possibly regulated by m6A were shared between the brain and gut, indicating that m6A regulated genes have significant organ specificity. In Zhang et al. study, it is predicted that the vast majority of genes regulated by m6A may have a negative regulatory relationship with gene expression, meaning that when m6A modification is high, gene mRNA expression is low, which is related to the promotion of mRNA degradation by m6A modification. In brain tissue, genes regulated by m6A exhibit m6A hypermethylation (low gene expression) in the hypothalamus, but m6A hypomethylation (high gene expression) in the cerebellum. In contrast, except for the duodenum, the m6A modification and gene expression variations of these genes in different parts of the intestinal tissue are relatively small. Moreover, these genes may be regulated by m6A in brain tissue are mainly enriched in the neuronal system and pathways related to neuronal ion channels; In intestinal tissue, it was found that genes that may be regulated by m6A are mainly enriched in the immune system and metabolism related pathways, which are consistent with the specific biological functions of intestinal tissue (digestion and immune function), further emphasizing the organ specificity of m6A in regulating gene expression. In this study, samples from both NEC and control groups were derived from ileal tissues to avoid variations in m6A modifications caused by tissue differences. This study conducted a correlation analysis between RNA seq and MeRIP seq to explore genes in NEC that may be regulated by m6A. The results showed that a significant number of regulated genes were expressed as RNA_up_MeRIP_up genes, indicating that m6A modification increases mRNA stability; Next is the RNA-down_MeRIP_up genes, indicating that m6A modification promotes mRNA degradation and reduces mRNA expression. The above results indicate that m6A modification has a tendency towards selectivity and regulatory complexity towards target genes. This study further analyzed the biological functions of genes regulated by possible m6A modifications in NEC.

NF-κB, a key regulator of inflammatory responses, serves as a central hub integrating multiple inflammatory signaling pathways (25). (26) demonstrated that Git2 deficiency promotes myeloid-derived suppressor cell (MDSC) recruitment in the intestinal tract through the NF-κB-CXCL1/CXCL12 axis, thereby ameliorating NEC. Furthermore, β-glucan has been shown to prevent NEC in neonatal mice by suppressing the TLR4/NF-κB signaling pathway, enhancing intestinal barrier function, and modulating gut microbiota composition (27). In this study, KEGG pathway analysis showed that genes possibly regulated by m6A were mainly enriched in cytokine cytokine receptor interactions, TNF signaling pathway, interactions between viral proteins and cytokines and their receptors, adhesive plaques, and NF-κB signaling pathway. The RNA_up_MeRIP_up gene is mainly related to the NF-κB signaling pathway. Previous studies have shown that m6A modification is enriched in the 3’UTR region of nuclear NFKBIA mRNA, promoting the degradation of NFKBIA mRNA. In METTL14 knockout intestinal stem cells, m6A deficiency on NFKBIA mRNA leads to upregulation of NFKBIA mRNA expression and enhanced protein expression, thereby inhibiting anti-apoptosis of NF-κB pathway, promoting TNF pathway induced cell death, and ultimately leading to severe colitis (10). It is speculated that the NF-κB signaling pathway in NEC may be regulated by m6A modification, but the specific regulatory mechanism is unclear and further research is needed for verification. Targeting the m6A modification process to regulate the NF-κB signaling pathway may be a target for preventing and treating NEC.

Previous studies have shown that CXCR4 is highly expressed in colitis. The use of CXCR4 antagonists can alleviate dextran sulfate sodium (DSS) - induced colitis, reduce inflammatory infiltration of colon tissue, downregulate the expression of TNF - and IFN - γ (28), and improve intestinal barrier function by regulating the expression of tight junction protein claudin (29). CXCR4 is a specific receptor that recognizes CXCL12, and studies have shown that the CXC12/CXCR4 pathway plays an important role in intestinal inflammatory diseases (30). (31) found that the concentrations of CXCR4 and CXCL12 in the plasma and peritoneal lavage fluid of children with NEC were significantly higher than those in children without intestinal inflammation. However, the mechanism of CXCR4 pathway in NEC is unclear, and the interaction mechanism between CXCL12 and CXCR4 in NEC still needs further research to confirm. (32) found that silencing METTL14 in human umbilical vein endothelial cells can downregulate mRNA expression, m6A modification on mRNA, and protein expression of CXCR4, suggesting that METTL4 promotes m6A modification on CXCR4 mRNA to make CXCR4 mRNA more stable, and the METTL14/CXCR4 axis can exacerbate TNF-induced inflammation in endothelial cells. This study analyzed the association between MeRIP seq and RNA seq, and found that CXCR4 belongs to the RNA_up_MeRIP_up gene. IHC staining confirmed that CXCR4 is highly expressed in NEC intestinal tissue, suggesting that m6A modification may increase the stability of CXCR4 mRNA, reduce degradation, and increase the expression level of CXCR4 protein. The above results suggest that the activation of the CXCR4 pathway is involved in the occurrence and development of NEC, and m6A modification may be an epigenetic regulatory mechanism for the abnormal activation of the CXCR4 signaling pathway in NEC. However, this study has several limitations. The precise regulatory role of m6A modification on the CXCR4 signaling pathway (e.g., whether it acts through stabilizing its mRNA or influencing its translation process) requires further mechanistic validation in cell-specific models. Future studies should focus on elucidating the molecular mechanisms of methyltransferase-m6A-CXCR4 axis in NEC.

In conclusion, this study utilized a murine NEC model to demonstrate increased m6A modification levels and elevated methyltransferase (METTL3/METTL14) expression in NEC intestinal tissues. Then, we mapped the first human m6A landscape in the ileum of NEC, and our results identified potential target genes and signaling pathways modulated by m6A, revealing that m6A modification may contribute to NEC pathogenesis through CXCR4 signaling regulation. Targeting the m6A modification process represents a potential new therapeutic target for NEC prevention and treatment. However, the study did not fully elucidate the precise molecular mechanisms of methyltransferase-m6A-CXCR4 axis in NEC.

## Data Availability

The human sequencing data are not publicly available due to data protection regulations. Supporting data are available from the corresponding author upon reasonable request.
